# IDH-mutant glioma risk stratification via whole slide images: Identifying pathological feature associations

**DOI:** 10.1016/j.isci.2024.111605

**Published:** 2024-12-16

**Authors:** Xiaotao Wang, Zilong Wang, Weiwei Wang, Zaoqu Liu, Zeyu Ma, Yang Guo, Dingyuan Su, Qiuchang Sun, Dongling Pei, Wenchao Duan, Yuning Qiu, Minkai Wang, Yongqiang Yang, Wenyuan Li, Haoran Liu, Caoyuan Ma, Miaomiao Yu, Yinhui Yu, Te Chen, Jing Fu, Sen Li, Bin Yu, Yuchen Ji, Wencai Li, Dongming Yan, Xianzhi Liu, Zhi-Cheng Li, Zhenyu Zhang

**Affiliations:** 1 Department of Neurosurgery, The First Affiliated Hospital of Zhengzhou University, Zhengzhou, Henan, China; 2 Department of Pathology, The First Affiliated Hospital of Zhengzhou University, Zhengzhou, Henan, China; 3 Institute of Basic Medical Sciences, Chinese Academy of Medical Sciences and Peking Union Medical College, Beijing 100730, China; 4 Department of Neurosurgery, Henan Provincial People’s Hospital, Zhengzhou, Henan, China; 5 Institute of Biomedical and Health Engineering, Shenzhen Institutes of Advanced Technology, Chinese Academy of Sciences, Shenzhen, China; 6 The Key Laboratory of Biomedical Imaging Science and System, Chinese Academy of Sciences, Shenzhen, China

**Keywords:** Medical imaging, Bioinformatics, Cancer

## Abstract

This article aims to develop and validate a pathological prognostic model for predicting prognosis in patients with isocitrate dehydrogenase (IDH)-mutant gliomas and reveal the biological underpinning of the prognostic pathological features. The pathomic model was constructed based on whole slide images (WSIs) from a training set (*N* = 486) and evaluated on internal validation set (*N* = 209), HPPH validation set (*N* = 54), and TCGA validation set (*N* = 352). Biological implications of PathScore and individual pathomic features were identified by pathogenomics set (*N* = 100). The WSI-based pathological signature was an independent prognostic factor. Incorporating the pathological features into a clinical model resulted in a pathological-clinical model that predicted survival better than either the pathological model or clinical model alone. Ten categories of pathways (metabolism, proliferation, immunity, DNA damage response, disease, migrate, protein modification, synapse, transcription and translation, and complex cellular functions) were significantly correlated with the WSI-based pathological features.

## Highlights

• A pathomics model was validated to stratify risk in IDH-mutant glioma patients using WSI
• We uncovered the biological significance of pathological features with prognostic value

## Graphical abstract

**Figure:**
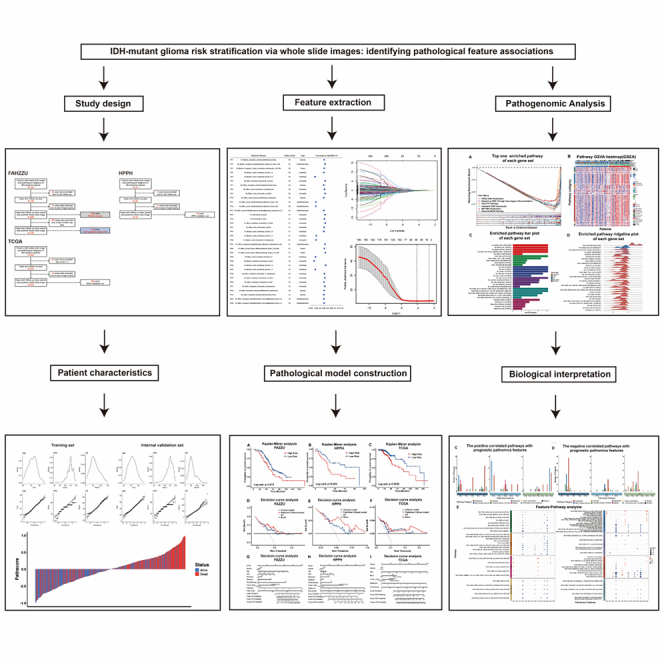

## Introduction

Gliomas represent the most prevalent form of primary malignant tumors in the adult central nervous system, accounting for approximately 46% of intracranial tumors.^1^ Despite continuous improvements in treatment regimens, including surgical resection followed by chemoradiotherapy and temozolomide chemotherapy, the incidence and mortality rates of gliomas have shown little reduction.^2^^,^^3^

Since the isocitrate dehydrogenase (IDH) mutations were found in a group of diffuse gliomas, the pathological diagnosis of glioma has entered the era of molecular pathology.^4^ The 2016 World Health Organization (WHO) Classification of Central Nervous System Tumors first clarified the role of IDH mutations in the diagnosis and classification of infiltrative diffuse gliomas.^5^ In the 2021 WHO Classification of Tumors of the Central Nervous System, a significant advancement was made by separately classifying adult and pediatric gliomas based on their distinct molecular pathogenesis and prognosis.^6^ Furthermore, adult-type diffuse gliomas were classified into three types mainly based on the status of IDH mutations and 1p/19q co-deletion: astrocytoma with IDH mutations, oligodendroglioma with both IDH mutation and 1p/19q co-deletion, and glioblastoma with IDH wild-type status.^7^ This classification system provides a more nuanced understanding of diffuse gliomas and their prognosis. IDH wild type generally implies a poor prognosis, whereas patients with IDH-mutant gliomas usually have longer survival times.^8^

In the past, histopathological diagnosis through visual examination of hematoxylin and eosin (H&E)-stained tissue sections under a microscope has been the gold standard for glioma classification.^9^ However, histopathological diagnosis of gliomas is a labor-intensive process and time-consuming process, as it requires pathologists to meticulously examine images of both coarse and fine resolution from a large number of tissue samples.^10^ In addition, complex classification criteria require more experienced pathologists to conduct thorough analyses.^11^ Despite well-established classification strategies, analyzing the same sample by multiple pathologists can easily lead to inconsistencies, as they may be influenced by various prejudices and draw conclusions from different angles or interpret complex classification criteria in different ways, which may lead to different conclusions.^12^ Subjective differences among observers can lead to misleading clinical decision-making and can impact subsequent treatment strategies. Therefore, there exists an urgent need to introduce an objective method for analyzing the pathological features of gliomas that assist human pathologists in practice. In recent years, the development of slide scanners has made it possible to digitize slides into images, a process known as whole slide imaging (WSI).^13^^,^^14^^,^^15^ Advances in scanning technology over the past 5 years have made it possible to scan large numbers of slides, paving the way for computational pathology.^14^ The development of computational pathology includes the study of artificial intelligence to assist pathologists in enhancing the efficiency and reliability of diagnoses.^16^^,^^17^ However, there are few studies on glioma in the field of pathomics and even fewer studies combining cancer pathomics with genomics. To our knowledge, the pathomics-genomic link of glioma has not been reported before.

This pathogenomics study aimed to (1) develop and validate a WSI-based model for predicting overall survival (OS) of patients with IDH-mutant gliomas and (2) investigate the biological underpinning of the prognostic pathological features by identifying underlying biological pathways using paired WSI and RNA sequencing data.

## Results

### Patient characteristics

The demographic details and clinical attributes of the participants are meticulously compiled in Table S4. To evaluate the data distribution within the training and internal validation sets, the Shapiro-Wilk test was utilized. This analysis indicated that key continuous variables, specifically age, Karnofsky performance status (KPS), and overall survival (OS), did not adhere to a normal distribution, as evidenced by *p* values less than 0.05. These findings are thoroughly documented in Figure S2 and Table S5. Given the nonnormal distribution of the continuous variables (age, KPS, and OS), the Wilcoxon rank-sum test was chosen for assessing distributional discrepancies between the training and internal validation sets. The analysis revealed no statistically significant variances in age, KPS, and OS across the two sets. Furthermore, the chi-squared test was employed to compare categorical variables such as gender, extent of tumor resection, receipt of radiation therapy, chemotherapy, tumor classification, and survival status between the sets. This test confirmed the absence of significant differences in these parameters, underlining the homogeneity between the training and internal validation sets in terms of both continuous and categorical variables.

### Pathological model construction, validation, and its incremental prognostic value

**Figure 1:**
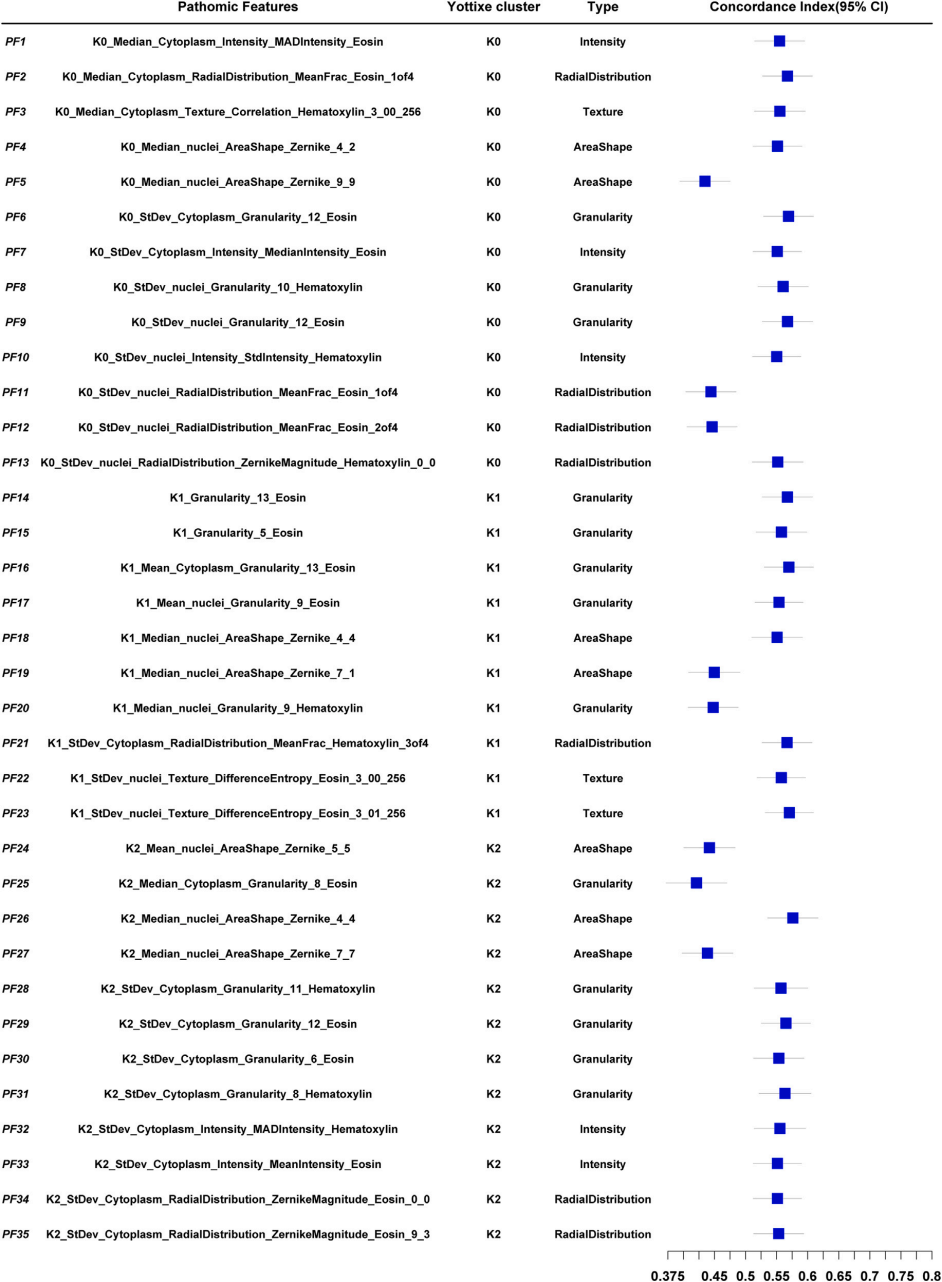
Brief information on selected pathological features Forest plot shows name, Yottixel cluster, type, and univariable prognostic performance in terms of concordance index (C index) for each of the 35 selected pathological features. Data are represented as C-index ± 95% confidence interval (CI), calculated using Noether’s method. The lower and upper bounds of the CI are derived based on the standard error (SE) of the C-index under the assumption of an asymptotic normal distribution.

*Pathological model construction:* a two-step process for image feature screening was performed. After the univariate Cox regression analysis, 195 of 6,456 features remained. Then, 35 features, PF1–PF15 selected by LASSO were used to calculate the PathScore as follows: PathScore = 0.10176 × PF1 + 0.10548 × PF2 − 0.01209 × PF3 + 0.20456 × PF4 − 0.04891 × PF5 + 0.016637 × PF6 − 0.13902 × PF7 + 0.02354 × PF8 + 0.05222 × PF9 + 0.07494 × PF10 − 0.00009 × PF11 − 0.14662 × PF12 + 0.01373 × PF13 + 0.16450 × PF14 + 0.02904 × PF15 − 0.01323 × PF16 + 0.05233 × PF17 + 0.03010 × PF18 − 0.14650 × PF19 − 0.08640 × PF20 + 0.13079 × PF21 + 0.07344 × PF22 + 0.02826 × PF23 − 0.06502 × PF24 − 0.13512 × PF25 + 0.14469 × PF26 − 0.03821 × PF27 + 0.14646 × PF28 + 0.12774 × PF29 + 0.04918 × PF30 + 0.13198 × PF31 + 0.08811 × PF32 − 0.00008 × PF33 − 0.02512 × PF34 − 0.17551 × PF35. The characteristics of the 35 pathomic features that make up the PathScore are shown in Figures 1 and 4B.

Details of the LASSO Cox model are shown in Figures S3 and S4. The features of PF1–PF35 are shown in Figure 1 and Table S6. According to a pathological training-set-based cutoff value determined by using R package “survminer,” patients were stratified into low-risk and high-risk groups, as shown in Figure S5.

#### Pathological model validation

**Figure 2:**
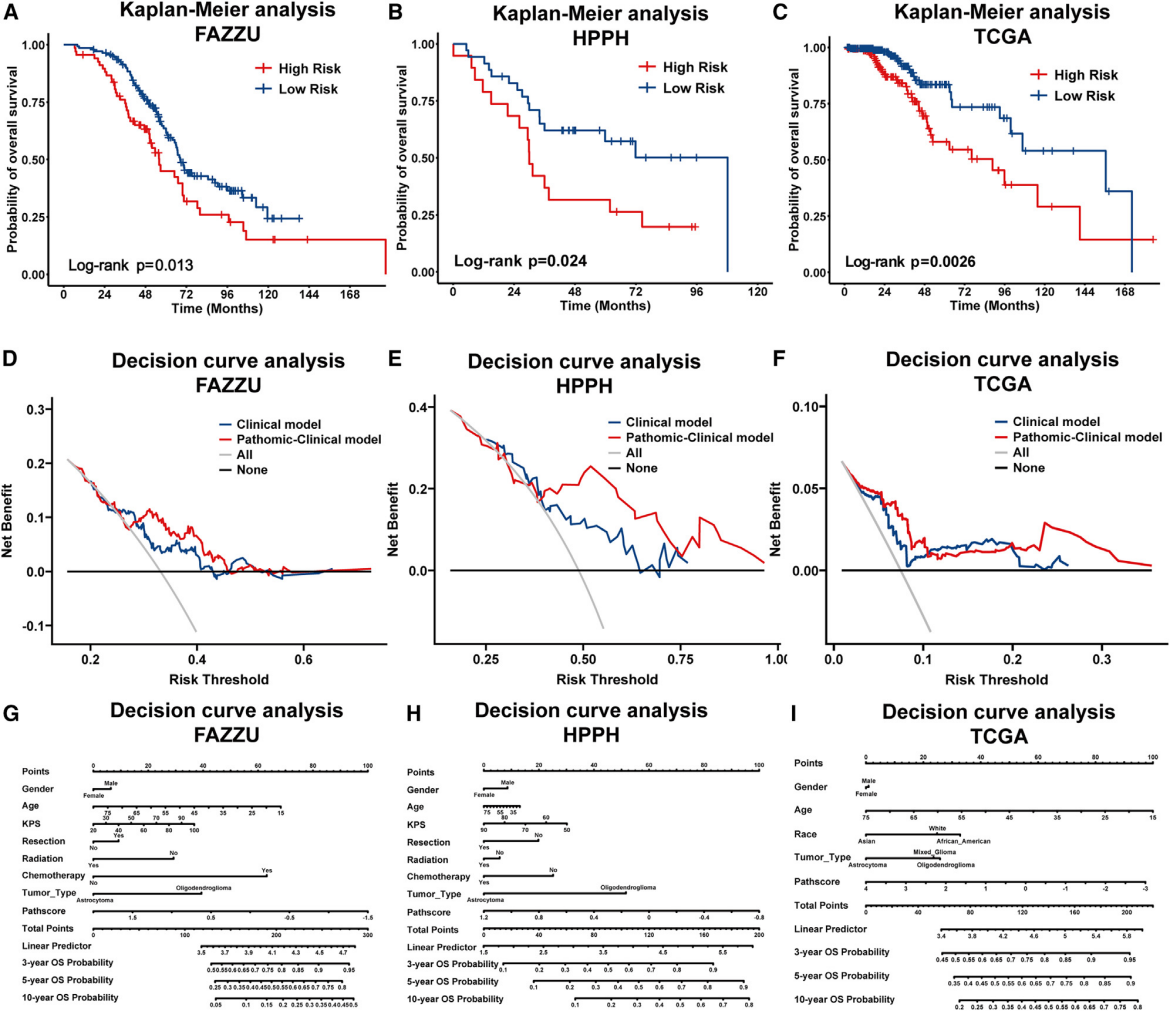
Internal and external validation of the pathological model (A–C) Kaplan-Meier curves for patients stratified by the PathScore in the internal and external validation sets. (D–F) Decision curve analysis for pathomic-clinical model nomogram and clinical model nomogram to estimate the OS in the internal and external validation sets. The x axis represents the threshold probability, and the y axis measures the net benefit. (G–I) The clinical model nomogram and the pathomic-clinical model nomogram for predicting the 3-, 5-, and 10-year OS in the internal and external validation sets.

As shown by Kaplan-Meier curves in Figures 2A–2C, the PathScore was significantly associated with OS in the internal validation set (log rank *p* = 0.013; hazard ratio [HR] = 0.627, 95% confidence interval [CI]: 0.416, 0.946), HPPH validation set (log rank *p* = 0.024; HR = 0.458, 95% CI: 0.212, 0.987), and TCGA validation set (log rank *p* = 0.0026; HR = 0.449, 95% CI: 0.263, 0.764). Multivariate Cox analysis demonstrated that the PathScore was an independent risk factor in the internal validation set (HR = 1.427; 95% CI: 1.019, 1.967; *p* < 0.039), HPPH validation set (HR = 2.540; 95% CI: 1.246, 5.175; *p* = 0.010), and TCGA validation set (HR = 1.417; 95% CI: 1.070, 1.877; *p* = 0.015). Table S7 shows more specific content.

#### Assessment of the incremental value of the pathological signature

The nomograms incorporating the clinical model (CM) or P-CM for OS prediction are shown in Figures 2G, 2H, and 2I, respectively. Compared with the CM nomogram, the P-CM nomogram showed significantly better agreement. Table 1 demonstrates the C-index and AIC values for the pathological model, CM, and P-CM in the training, internal validation, and external validation sets. The decision curves of the internal and external validation sets, illustrated in Figures 2D–2F, show the clinical usefulness of the P-CM.

**Table 1:** C-indices and AIC values for OS prediction in training and validation datasets

Model	C-index (95% CI)	AIC
**Training Set** **OS**		

Pathomic model	0.719 (0.687–0.751)	4634.315
Clinical model	0.679 (0.644–0.720)	4633.429
Pathomic-clinical model	0.754 (0.725–0.783)	4592.599

**Internal Validation Set** **OS**		

Pathomic model	0.573 (0.518–0.628)	2003.063
Clinical model	0.604 (0.547–0.661)	2006.124
Pathomic-clinical model	0.640 (0.585–0.694)	2002.640

**HPPH Validation Set**		

Pathomic model	0.630 (0.551–0.708)	516.992
Clinical model	0.650 (0.556–0.744)	522.400
Pathomic-clinical model	0.676 (0.586–0.756)	520.878

**TCGA Validation Set**		

Pathomic model	0.620 (0.526–0.714)	3379.531
Clinical model	0.688 (0.614–0.763)	3370.382
Pathomic-clinical model	0.727 (0.660–0.793)	3372.380

Data are represented as C-index ± 95% confidence interval (CI), calculated using Noether’s method. The lower and upper bounds of the CI are derived based on the standard error (SE) of the C-index under the assumption of an asymptotic normal distribution.

#### Pathogenomic analysis: GSEA

**Figure 3:**
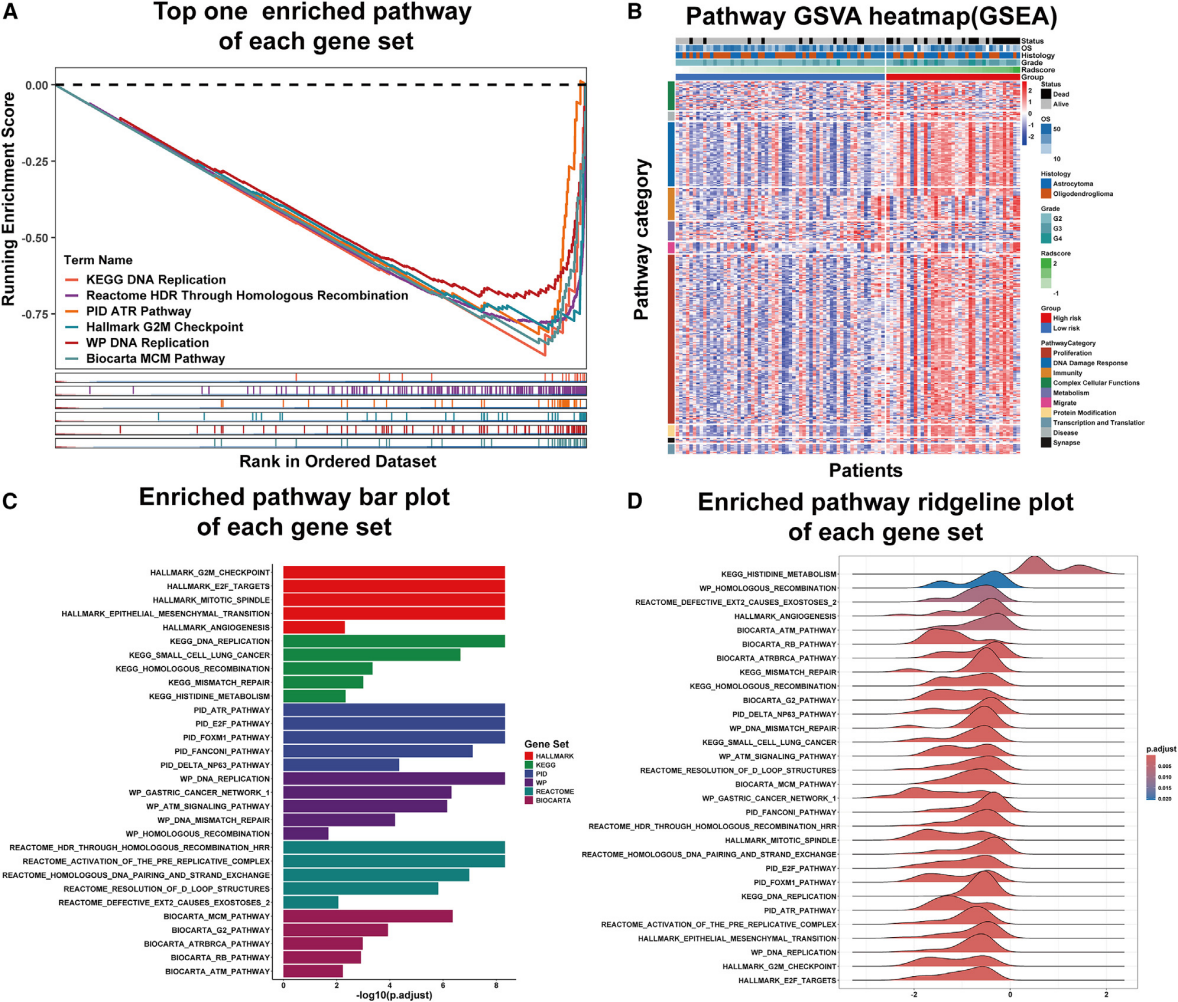
Results of gene set enrichment analysis (A) Top enriched pathway in Kyoto Encyclopedia of Genes and Genomes (KEGG), Hallmark, Reactome, BioCarta, Pathway Interaction Database (PID), and WikiPathways. (B) A heatmap of the gene set variation analysis (GSVA) score of GSEA pathways significantly correlated with the PathScore. (C) Bar plot of the top enriched pathways in each gene set. (D) Ridgeline plot of the top enriched pathways in each gene set.

**Figure 4:**
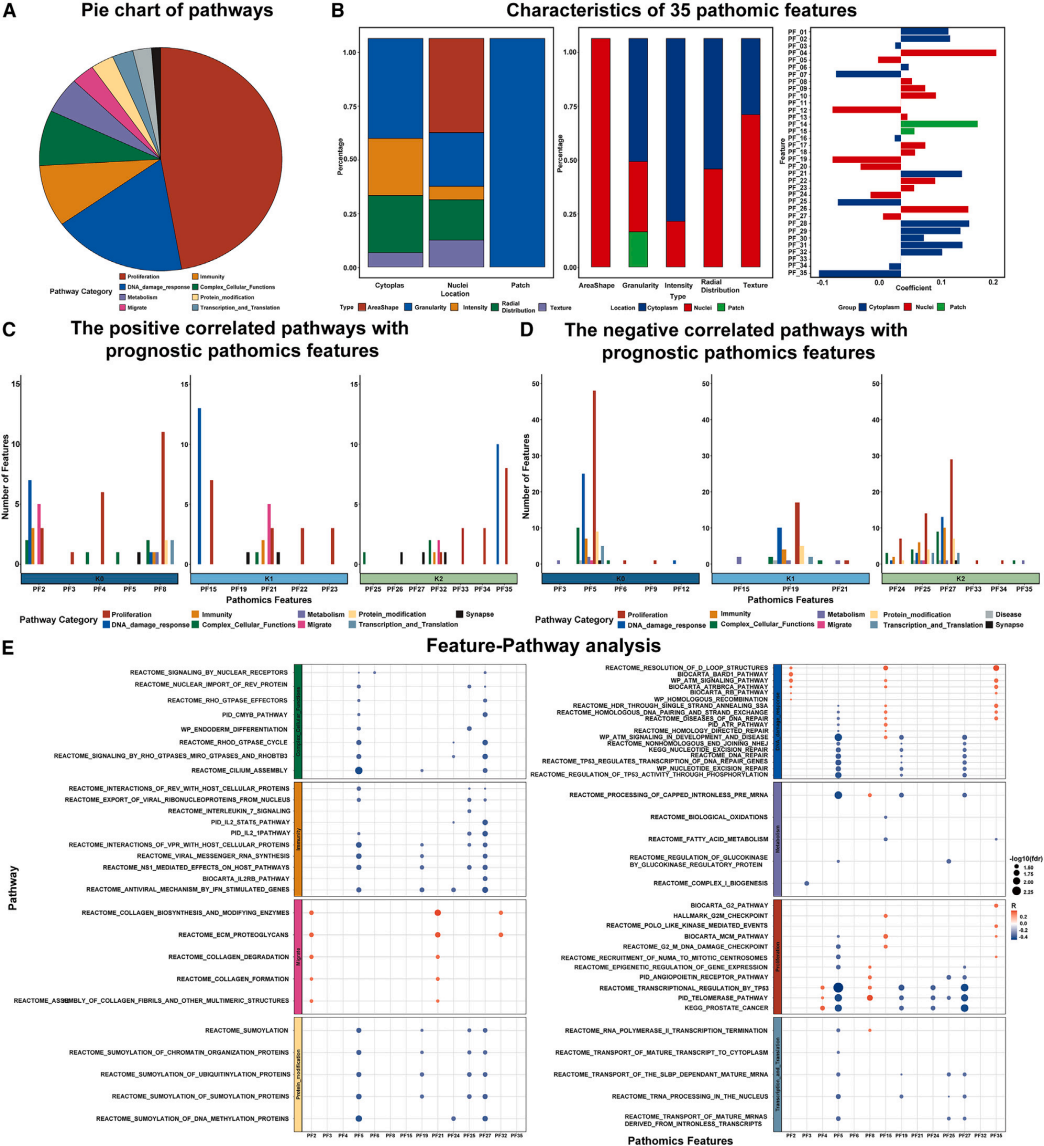
Pathogenomics linking between 35 pathological features constituting the PathScore and their significantly associated pathways (A) Categories of final pathways. (B) Characteristics of 35 pathomic features. (C) The number of positively correlated pathway species corresponding to each prognostic pathological feature. (D) The number of negatively correlated pathway species corresponding to each prognostic pathological feature. (E) A bubble plot of correlation between prognostic pathological features and classic biological pathways.

First, we identified 606 pathways through the application of GSEA. Second, the GSVA score of these pathways and PathScore were subjected to Pearson correlation analysis. This screening process resulted in the selection of 324 pathways that exhibited a false discovery rate (FDR) <0.05 and Pearson correlation coefficient >0.3 (positive association) or < −0.3 (negative association). These pathways were then classified into 10 categories: metabolism, proliferation, immunity, DNA damage response, disease, migrate, protein modification, synapse, transcription and translation, and complex cellular functions, as shown in Figure 4A and Table S8. Figure 3A illustrates the top enriched pathway within each gene set. Additionally, a heatmap depicting the GSVA scores of the GSEA pathways within the pathogenomics set is presented in Figure 3B and Table S9. The top enriched pathways in each gene set are shown in Figures 3C and 3D.

### Biological interpretation of the pathological features

First, the correlation between the individual prognostic pathological feature (*N* = 35) and 324 pathways was evaluated using Pearson correlation analysis. The numbers of pathways that significantly correlated with the individual pathological feature are shown in Figures 4C and 4D. Representative pathways that were significantly correlated with the prognostic pathological features are presented in Figure 4E.

## Discussion

Accurately predicting prognosis plays a pivotal role in the clinical risk stratification and management of patients with IDH-mutant gliomas.^18^^,^^19^ This study represented an effort toward precise stratification on adult IDH-mutated glioma patients, leveraging quantitative pathological features extracted from WSIs. Our research contributes a comprehensive biological interpretation of these pathological features. It involved a semi-automatic workflow capable of identifying thousands of pathological features from the WSIs by combining the Yottixel and Cellprofiler. Furthermore, to reinforce the robustness of our findings, we conducted additional validations of our pathological model using histopathological images and clinical data obtained from another independent institution.

There are a few factors that play a crucial role in pathological prognostic modeling. First, the adoption of convenient image processing methods is essential for extracting quantitative pathohistological features. Currently, there exists no unanimous agreement on the extraction of such features. Notably, CellProfiler, a free and open-source software, offers automated measurement of biological image phenotypes and has demonstrated satisfactory performance in digital pathology analysis.^20^^,^^21^ Second, a pragmatic machine learning method is imperative for selecting prognostic features. To address this, we opted for the LASSO-Cox regression model.^22^ Widely employed in various clinical practices, including tumor prognosis and cardiovascular disease prognosis, this model excels in its robust capability to handle high-dimensional data.^22^^,^^23^ A notable application of this is evident in the realm of breast cancer diagnosis, where pathological serves to distinguish between carcinoma *in situ* and invasive carcinoma, while also providing insights into patient prognosis.^24^ Simultaneously, pathological technology shows its formidable capability in forecasting disease prognosis by scrutinizing molecular changes throughout the progression of the ailment. In the context of lung cancer prognosis, for instance, pathological proves instrumental in predicting a patient’s likelihood of recurrence and overall survival.^25^^,^^26^

However, the wealth of information encapsulated in whole slide pathology images posed a substantial computational challenge for researchers. The sheer scale of the original images made them highly intricate to manipulate, and informatics workflows requiring manual tumor tissue segmentation became impractical when dealing with millions of image tiles.^27^ As a consequence, earlier investigators had to invest considerable time and effort in manually selecting representative views within the tissue.

Deep learning technologies are widely applied in pathology research, offering significant advancements in various areas.^28^ For instance, in the automatic segmentation of pathological slides, deep learning models can accurately identify and segment different tissue types and cellular structures within the slides, enabling pathologists to analyze samples more quickly and precisely.^29^^,^^30^ Moreover, these models excel in the automated analysis, detection, and classification of various cancer types.^31^^,^^32^ In the case of breast cancer, prostate cancer, and lung cancer, deep learning algorithms have demonstrated diagnostic accuracy comparable to, and sometimes even surpassing, that of human pathologists.^33^^,^^34^^,^^35^ Additionally, deep learning models can predict patient outcomes by analyzing microscopic features within pathological images.^36^ By identifying image characteristics associated with poor prognosis, these models offer more detailed and nuanced prognostic assessments compared to traditional methods.^37^ Nevertheless, the biological implications of these pathologic features have seldom been-investigated, presenting notable obstacles to the clinical dissemination of pathohistological models.^38^

This study differs from previous pathology studies in several ways. This study has two aspects that may contribute to the current research on cancer pathomics. Primarily, it introduces the application of the Yottixel digital pathology processing engine, streamlining the comprehensive processing of WSIs encompassing tasks such as tissue identification, clustering, and segmentation.^39^ This methodology notably amplifies efficiency and robustness. In contrast to earlier studies, this study relies on manual screening for feature extraction in WSI preprocessing. Requiring minimal human intervention, except for patch selection and model training, it holds the potential to predict survival outcomes rapidly and impartially for a myriad of patients. Furthermore, this study delves into the biological underpinnings of pathologic features from a multifaceted perspective. Initially, we elucidate the biological drivers of PathScore, followed by a systematic exploration of the categories of biological pathways underpinning individual pathological features and their corresponding distributions. Specifically, we delved into the categories and quantity of biological pathways associated with each individual pathological feature.

From a locational standpoint, PathScore integrates 17 nuclear features and 16 cytoplasmic features. When considering the characteristics of these features, the distribution includes 6 Intensity features, 9 RadialDistribution features, 4 Texture features, 6 AreaShape features, and 10 Granularity features. A comprehensive analysis that synthesizes both feature location and property reveal that nuclear characteristics are predominantly influenced by AreaShape, Granularity, and Texture, whereas cytoplasmic characteristics are chiefly associated with Granularity, RadialDistribution, and Intensity. These features provide a detailed characterization of attributes such as size, shape, intensity, granularity, and texture of both the cell nucleus and cytoplasm. Existing literature indicates that nuclear size and shape are indicative of cellular proliferation status, and texture features offer insights into chromatin structure. The intensity distribution sheds light on nucleolar distribution within the nucleus. Cytoplasmic granularity is indicative of cellular metabolic activity, whereas texture features highlight organelle distribution.^40^^,^^41^^,^^42^ Radial distribution features of the cytoplasm reflect the spatial distribution of proteins within the cytoplasm.^43^

Further pathogenomic analysis of individual pathological features showed that the PF2, PF15, and PF35 were mainly positively correlated with the DNA damage response pathways, and these features were mainly radial signal distribution intensity in the cytoplasm. The PathScore coefficients of these features were mainly positive, indicating that higher signature intensity may be associated with upregulation of DNA damage pathways and poorer prognosis. In contrast, PF5, PF19, and PF27 were negatively correlated with DNA damage pathway, and these features were mainly nuclear morphology features. Their PathScore coefficients are all negative, indicating that higher feature strength may be associated with downregulation of DNA damage pathways and better prognosis. These two types of features mainly reflect the distribution of cytoplasmic proteins and the state of cell proliferation.

In a focused examination of the predominant biological pathway in glioma, specifically the proliferation pathway, it was observed that the PF4, PF8, PF15, and PF35 features exhibit a positive correlation with the activity of these pathways. These features are mainly the morphology and granularity of the cell nucleus, and their coefficients are mostly positive. This means that the enhanced signal intensity in these features will lead to increased activation of the proliferation pathway, resulting in poor patient prognosis. In essence, these features together reflect the activated proliferation state of the nucleolar chromatin in the cell nucleus. In contrast, features such as PF5, PF19, PF24, and PF27 are negatively correlated with proliferation pathways. They are mainly the shape characteristics of the cell nucleus, and their coefficients are all negative, which means that the higher the intensity of these features, the less the activation of the proliferation pathway will be, thus giving patients a relatively better prognosis.

In addition, we noticed that the features positively correlated with distant tumor metastasis pathways were mainly PF2, PF21, and PF32. They were mainly radial distribution and intensity characteristics of the cytoplasm, and the coefficients were all positive, which reflected the distribution of proteins in the cytoplasm. It also means that higher feature expression is associated with highly activated migration pathways and poorer prognosis.

Pathological features that are positively correlated with immune pathways, such as PF2, PF21, and PF32, are mainly radial distribution features of the cytoplasm, and the coefficients are all positive, which means that when the intensity of this part of the pathological features is greater, the immune activity is more intense, but the patient’s prognosis is worse. In contrast, the PF5, PF19, PF25, and PF27 features are mainly morphological features of the cell nucleus. They are negatively correlated with immune pathways, and the coefficients are all negative, which means that when the intensity of their features is greater, the immune activity is weaker, but the patient’s prognosis is relatively good. This immune activity may indicate immune escape, making tumor cells more resistant and invasive, leading to a poorer prognosis.

From the perspective of pathomics feature, there are several features, such as PF5, PF19, PF25, and PF27, that are negatively correlated with the upregulation of almost all types of biological pathways, that is, the feature intensity is negatively correlated with the pathway activity. The PathScore coefficients of these features are exactly negative, indicating that higher feature intensity may be related to reduced pathway activity. Downregulation of activity is associated with better prognosis. It mainly represents the area, shape, and internal fine-grained characteristics of the nucleus, as well as the fine-grained characteristics of the cytoplasm. This means that they reflect the proliferation and metabolic status of cells.

Our investigation underscores the intricate interplay of biological pathways shaping individual prognostic pathological features. Notably, these features demonstrate associations with diverse categories of biological pathways, rather than being confined to a single pathway or pathway category. This multifaceted relationship sheds new light on therapeutic strategies derived from biologically interpretable pathological models. For instance, our findings reveal that the high-risk group identified by our pathological model correlates significantly with various malignant tumor processes, including immunity, proliferation, cellular biological functions, DNA damage response, and metabolism. Consequently, we propose that therapeutic interventions aimed at anti-cell proliferation and immunosuppression may hold promise in individuals characterized by elevated PathScore, potentially enhancing treatment efficacy.

In summary, this pathogenomic study demonstrated that prognostic pathological features derived from WSI are driven by distinct pathways involved in metabolism, proliferation, immunity, DNA damage response, disease, migrate, protein modification, synapse, transcription and translation, and complex cellular functions. The proposed biologically explainable pathological model may have the potential to inform therapeutic strategies for IDH-mutant gliomas.

### Limitations of the study

Our study has several limitations. First, this was a retrospective study; future prospective multi-center studies are required to further corroborate our pathogenomic findings. Second, although the current study revealed the biological underpinning of WSI-based pathological features at transcriptomic level, future biological validations at protein and *in vivo* levels are required to confirm the findings. Third, this study mainly used correlation analysis methods for biological interpretation of pathological omics features. Although the statistical results show a close relationship between pathological features and certain biological activities, we would like to emphasize that the results of correlation analysis do not mean a clear causal relationship between the two variables. Reliable biological interpretations need to be verified by animal experiments or mechanism studies.

## Resource availability

### Lead contact

Further information and requests for resources and reagents should be directed to and fulfilled by the lead contact, Zhenyu Zhang (fcczhangzy1@zzu.edu.cn).

### Materials availability

The glioma tissues in our study come from our pre-existing mRNA sequencing dataset, which have been deposited in the Genome Sequence Archive database (HRA006184). No new and unique reagents were produced in this study. Pathological and clinical data cannot be made public because they contain personal information of patients.

### Data and code availability

• Data availability: the datasets used and/or analyzed during the current study available from the corresponding author on reasonable request.
• Code availability: the codes used and/or analyzed during the current study available from the corresponding author on reasonable request.
• Other items: all other items used and/or analyzed during the current study available from the corresponding author on reasonable request.

## Acknowledgments

This research was funded by the 10.13039/501100001809National Natural Science Foundation of China (grant numbers: 82273493, U20A2017, 82173090, and U1904148), the Natural Science Foundation of Henan Province for Excellent Young Scholars (grant number: 232300421057), the Science and Technology Program of Henan Province (grant numbers: 242102311107, 202102310136 and 212102310113), Henan Province Outstanding Young Talent Project in Health Science and Technology Innovation for Young and Middle-aged People (YXKC2022035), the Key project of Natural Science Foundation of Henan Province (232300421125), and the Joint Fund of Henan Province's Science and Technology Research and Development Program (242301420014).

All funders played no role in study design, data collection, analysis and interpretation of data or the writing of this manuscript.

## Author contributions

Z.Y.Z., Z.C.L., and X.Z.L. performed the research conception. X.T.W., Z.L.W., W.W.W., Z.Q.L., D.Y.S., Y.Q.Y., H.R.L., C.Y.M., M.M.Y., Y.H.Y., Z.Y.M., Y.G., D.L.P., W.C.D., Y.N.Q., M.K.W., T.C., W.Y.L., and J.F. S.L. performed the data acquisition. X.T.W., Z.L.W., and Z.Q.L. performed the data processing. X.T.W. and Z.L.W. performed the statistical analysis. Z.Y.Z., Z.C.L., and X.Z.L. performed the project administration. X.T.W., Z.L.W., W.W.W., Z.Q.L., and Z.Y.Z. performed the manuscript drafting. All authors have read and approved the final version of the manuscript.

## Declaration of interests

All authors declare no financial or non-financial competing interests. Funders did not play any part in study design, data collection, data analyses, interpretation, or writing of the manuscript.

## STAR★Methods

### Key resources table

**Table:** 

REAGENT or RESOURCE	SOURCE	IDENTIFIER
**Chemicals, peptides, and recombinant proteins**		

3 μL USER Enzyme	NEB	N/A
KAPA HiFi HotStart DNA Polymerase	Kapa Biosystems Inc	N/A
Shrimp Alkaline Phosphatase (SAP) enzyme	NEB, Ipswich	N/A
PCR reaction solution C	CWBIO, Beijing	N/A
natrium asceticism-ethanol mixture	3M	
Hi-Di™ Formamide	Thermo Fisher Scientific	

**Critical commercial assays**		

Qubit® RNA Assay Kit	Life Technologies	Qubit®2.0Flurometer#Q32852
NEBNext® UltraTM RNA Library Prep Kit for Illumina®	NEB	NEB #E7770L
AMPure XP system	Beckman Coulter	N/A
TruSeq PE Cluster Kit	Illumia	v3-cBot-HS
BigDye	hermo Fisher Scientific, Waltham	BigDye™ Terminator v3.1 Cycle Sequencing Kit
probes mixture	GP Medical Technologies	N/A

**Oligonucleotides**		

forward primer primers IDH1-F: 5′-CGGTCTTCAGAGAAGCCATT-3′, IDH1-R:5′-CACATTATTGCCAAC ATGAC-3′,IDH2-F:5′-AGCCCAT CATCTGCAAAAAC-3′,IDH2-R: 5′-CTAGGCGAGGAGCTCCAGT-3′	This paper	N/A

**Software and algorithms**		

Yottixel	Shivam Kalra et al.	https://github.com/RhazesLab/yottixel
CellProfiler 4.2	Anne E.Carpenter	https://cellprofiler.org/
R studio	Posit company	https://posit.co/downloads/
R project 4.4.1	Ross Ihaka and Robert Gentleman	https://www.r-project.org/
Chromas software	Technelysium, South Brisbane	https://technelysium.com.au/wp/chromas/#:∼:text=Chromas%20is%20a%20free%20trace%20viewer%20for%20simple%20DNA%20sequencing

**Other**		

MAGSCAN-NER scanner	KFBIO	KF-PRO-005
survminer	R package	https://github.com/kassambara/survminer
rmda	R package	https://github.com/mdbrown/rmda
DESeq2	R package	https://github.com/thelovelab/DESeq2
clusterProfiler	R package	https://github.com/YuLab-SMU/clusterProfiler
HTSeq v0.6.0	Python	https://github.com/htseq/htseq
The glioma tissues in our study come from our pre-existing mRNA sequencing dataset, which have been deposited in the Genome Sequence Archive database	This paper	Genome Sequence Archive database (HRA006184)

### Experimental model and study participant details

#### Study design

The study procedures are illustrated in the figure below, and consist of data collection, pathological model building, pathogenomic analysis, identification of pathological-related pathways, and biological interpretation of pathological features. First, we developed and validated a whole slide imaging (WSI)-based pathological model to predict the prognosis of patients with IDH-mutant gliomas. We then utilized gene set enrichment analysis (GSEA) to identify the biological pathways associated with pathological features. Third, the pathways associated with Pathscore were selected to reveal the biological implications of pathological features. Finally, the underlying biological basis of the individual pathological feature was revealed via Pearson correlation analysis.

**Figure:**
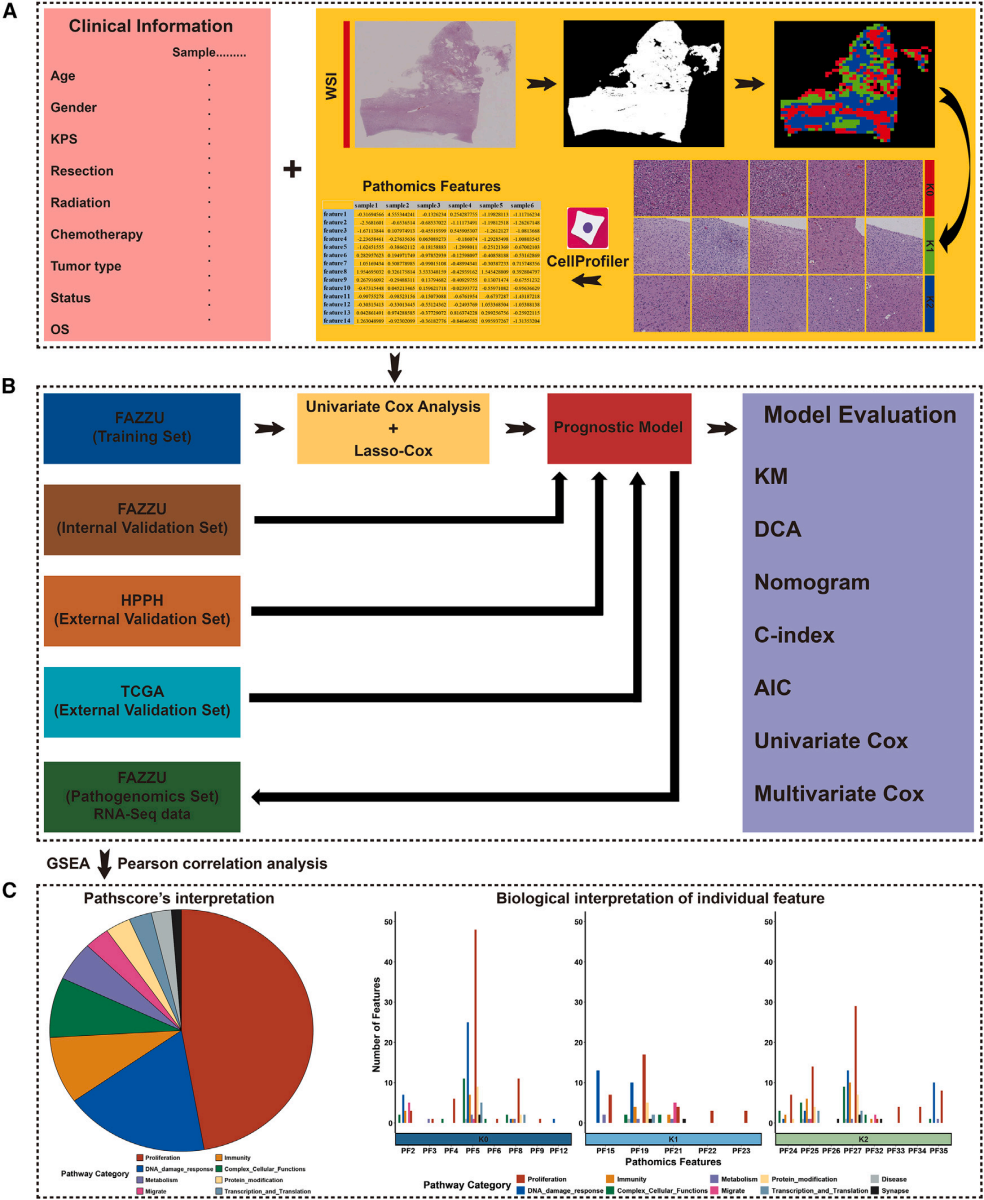
Workflow of this study (A) Collection of clinical data and preprocessing of pathological data. (B) The training and validation process of the prognostic model. (C) Annotating individual prognostic pathological feature.

#### Study cohorts

A total of 695 adult patients pathologically diagnosed with IDH-mutant gliomas and with WSIs at the First Affiliated Hospital of Zhengzhou University (FAHZZU) during 2011–2021 were enrolled in this study as the FAHZZU dataset. The FAHZZU dataset was divided into a training set (*N* = 486) and internal validation set (*N* = 209)，which is randomly sampled with a ratio of about 7:3, and the clinical parameters are balanced. Additionally, 100 patients from the FAHZZU dataset with RNA-seq data of fresh frozen tumor tissues were designated as the pathogenomics set. Besides, patients diagnosed with IDH-mutant gliomas and with WSI images at the Henan Provincial People’s Hospital (HPPH) during 2011–2022 were enrolled in this study as the HPPH external validation set (*N* = 54) to externally validate the reproducibility of the prognostic pathological model. At the same time, we also included relevant patients in the TCGA database (*N* = 352) for external validation. The inclusion and exclusion criteria are shown in Figure S1. Detailed information on RNA-seq, the detection of IDH mutations and detection of chromosome 1p/19q status is provided in Method S1.

#### Ethics statements

The Human Scientific Ethics Committee of the First Affiliated Hospital of Zhengzhou University (FAHZZU) and Henan Provincial People’s Hospital (HPPH) approved this study (approval numbers: 2019-KY-176, 2023-KY-1028 and 2023-174).

Informed Consent: Given the retrospective nature of this study, the committee waived the requirement for written informed consent. However, all fresh tumor tissue specimens used in this research were approved by the patients, with informed consent duly obtained.

### Method details

#### WSI data acquisition and preprocessing

Pathology slides were scanned at 20× magnification using a digital pathology scanner (KF-PRO-120-HI) to obtain the original whole slide images (WSI). Subsequently, the original WSI underwent color space conversion, tissue segmentation, patch selection, and feature extraction. Specifically, the WSI at the 5x resolution was converted from RGB to Lab color space, and Otsu’s algorithm was then applied to calculate a segmentation threshold for segmenting the tissue from the WSI. The obtained tissue image was tiled into many 1024 × 1024 patches at 20× magnifications, where these patches were adjacent to one another covering the WSI. A Python package Yottixel was used to select the optimal patches for further analysis. Finally, CellProfiler (v4.2.5) software was used to extract features from each selected patch.

In our study, one patient had one WSI. After excluding poor-quality images, 695 WSIs from FAHZZU, 54 WSIs from HPPH, 352 WSIs from TCGA were finally included for our study. Detailed information on pathology specimen preparation process is provided in Table S1.

#### Image preprocessing and pathological feature extraction

First, we used an image search engine named “Yottixel” for fully automated preprocessing of the original WSIs, including three steps: WSI tiling into 1024 × 1024 patches, classifying between tissue and non-tissue patches (patches containing less than 85% tissues were considered as non-tissue patches^44^), and K-means clustering of tissue patches based on their imaging patterns. Consequently, we obtained three clusters of patches for each patient: K0, K1 and K2, where patches within the same cluster were considered to have similar imaging patterns. Then, using the Yottixel tool, the top five patches with the best image quality within each cluster were calculated and selected.

Then, a tool named CellProfiler was used to extract the pathomics features from all the five selected patches within each cluster.^45^ The pipeline started with separating stains from background using the UnmixColors module. Then, the IdentifyPrimaryObjects, IdentifySecondaryObjects and IdentifyTertiaryObjects modules with adaptive Otsu thresholding were used to identify cell nuclei, cell bodies and cytoplasm. Then, a set of quantitative features can be calculated by using various modules such as Measure object size and shape, Measure texture, Measure Granularity, Measure Object Neighbors, Measure Area Occupied, Measure object intensity, and Measure object intensity distribution.^45^ The extracted quantitative pathological features can comprehensively characterize the histological images, such as nucleus and cytoplasm size, shape, texture, pixel intensity distribution, and proximity relationships. Note that here for each patient we had three clusters of patches, and for each cluster we had five patches. To well represent each cluster, we calculated the average value for each feature over the five patches within each cluster. Finally, we had 6456 features for each patient. Detailed information on pathomics features is provided in Tables S2 and S3.

#### Identification of driving pathways through GSEA analysis

Initially, we acquired Log2FoldChange values for each gene by conducting differential gene expression analysis across high-risk and low-risk groups, as determined by a pathological model.^46^ Following this, genes were ranked based on their Log2FoldChange values, and Gene Set Enrichment Analysis (GSEA) was applied to identify significantly enriched pathways, characterized by an FDR-adjusted hypergeometric *p*-value of less than 0.05. We further conducted a Pearson correlation analysis between the gene set variation analysis (GSVA) scores of these enriched pathways and the pathscore. Pathways that maintained an FDR <0.05 and Pearson correlation coefficient >0.3 (positive association) or < -0.3 (negative association) after this analysis were considered significant. Differential analysis was performed using the R package “DESeq2”.^47^ GSEA was performed using the R package “clusterProfiler”, querying the following annotated gene set databases: Kyoto Encyclopedia of Genes and Genomes, Hallmark, Reactome, BioCarta, Pathway Interaction Database, WikiPathways.^48^^,^^49^^,^^50^^,^^51^^,^^52^^,^^53^

#### Biological interpretation of pathological features

Our exploration into the biological pathways linked to specific pathological features involved performing Pearson correlation analysis between the prognostic pathological features and GSVA scores of pertinent pathways.^54^ Pathways that exhibited an FDR <0.1 were selected to elucidate the biological basis of each prognostic pathological feature, aiming to provide a clearer understanding of their implications.

### Quantification and statistical analysis

#### Pathological model construction and validation

We employed a two-step process to screen pathological features in the training set. Above all, we calculated the univariate concordance index (C-index) of the features to reflect the relationship between the pathological features and OS. Pathological features with a *p*-value <0.05 and univariate C-index ≥0.55 (positive association) or ≤0.45 (negative association) were retained for further analysis. Then, least absolute shrinkage and selection operator (LASSO) penalized Cox proportional hazards regression analysis was used to select dependable pathological features and construct the pathological model.^55^ Pathscore was calculated as a linear combination of features with their nonzero coefficients generated by LASSO. The R package “survminer” was used to calculate the Pathscore cutoff value for the training set.^56^ Then, the cutoff value was applied to the internal and external validation sets. The association between the Pathscore and OS was evaluated using Kaplan-Meier analysis. A log rank test was used to assess the survival difference, where a *p*-value <0.05 indicated a significant difference. Calibration curves were plotted to assess the agreement between predicted and observed survival. Decision curves were plotted to evaluate the clinical usefulness of the pathological-clinical model (P-CM). The C-index was calculated using the R package “survival” to measure the discrimination performance of the model.^57^ The Akaike information criterion (AIC) was computed using R package “stats” to assess the risk of model overfitting.^58^ Decision curve analysis was performed using the R package “rmda” to confirm the clinical usefulness of the P-CM. False Discovery Rate (FDR) correction method was applied during both the Gene Set Enrichment Analysis (GSEA) and correlation analysis to reduce the likelihood of false positives.

### Additional resources

The Human Scientific Ethics Committee of the First Affiliated Hospital of Zhengzhou University (FAHZZU) and Henan Provincial People’s Hospital (HPPH) approved this study (approval numbers: 2019-KY-176, 2023-KY-1028 and 2023-174).

## Supplemental information

Document S1. Figures S1–S5 and Tables S1, S2, and S4–S7 and Methods S1

Table S3. List of pathomics features extracted by the IDH-mutant gliomas image processing pipelin

Table S8. A summary of the PathScore-related GSEA-enriched pathways

Table S9. Classification of PathScore-related GSEA-enriched pathways
