# Supplementary material for: IDH-mutant glioma risk stratification via whole slide images: Identifying pathological feature associations
Source: iScience. 2024 Dec 16;28(1):111605. doi: 10.1016/j.isci.2024.111605 (PMC11751506; doi:10.1016/j.isci.2024.111605)
Supplement: Document S1. Figures S1–S5 and Tables S1, S2, and S4–S7 and Methods S1 [file mmc1.pdf]

iScience, Volume *28*

Supplemental information

IDH-mutant glioma risk stratification via whole slide images: Identifying pathological feature associations

Xiaotao Wang, Zilong Wang, Weiwei Wang, Zaoqu Liu, Zeyu Ma, Yang Guo, Dingyuan Su, Qiuchang Sun, Dongling Pei, Wenchao Duan, Yuning Qiu, Minkai Wang, Yongqiang Yang, Wenyuan Li, Haoran Liu, Caoyuan Ma, Miaomiao Yu, Yinhui Yu, Te Chen, Jing Fu, Sen Li, Bin Yu, Yuchen Ji, Wencai Li, Dongming Yan, Xianzhi Liu, Zhi-Cheng Li, and Zhenyu Zhang

Supplementary Material

This supplementary material includes:

Methods S1. Supplementary experimental method
Table S1. Details of Whole Slide Image Preparations
Table S2. Details of CellProfiler modules
Table S4. Characteristics of patients in training, internal validation
Table S5. Results of Shapiro-Wilk test for continuous variables in training and internal validation sets
Table S6. List of pathomics features used for modeling
Table S7. Cox regression analysis within the training, internal validation and external validation sets
Figure S1. The criteria for patients' inclusion and exclusion
Figure S2. Continuous variable normal test visual results
Figure S3. Pathomics feature selection 1
Figure S4. Pathomics feature selection 2
Figure S5. Pathscore for each patient

Methods S1 Supplementary experimental method

This part describes in detail the supplementary experimental methods related to the graph/table or STAR method mentioned in the main document, including the preparation and sequencing of RNA samples, the detection of IDH mutation and the detection of chromosome 1p/19q status by fluorescence in situ hybridization (FISH).

1.RNA samples preparation and sequencing

(1) RNA quantification and qualification: RNA degradation and contamination were monitored on 1% agarose gels. RNA purity was checked using the NanoPhotometer® spectrophotometer (IMPLEN, CA, USA). RNA concentration was measured using Qubit® RNA Assay Kit in Qubit® 2.0 Flurometer (Life Technologies, CA, USA). RNA integrity was assessed using the RNA Nano 6000 Assay Kit of the Bioanalyzer 2100 system (Agilent Technologies, CA, USA).
(2) Library preparation for Transcriptome sequencing: A total amount of 3 µg RNA per sample was used as input material for the RNA sample preparations. Sequencing libraries were generated using NEBNext® UltraTM RNA Library Prep Kit for Illumina® (NEB, USA) following manufacturer's recommendations and index codes were added to attribute sequences to each sample. Briefly, mRNA was purified from total RNA using poly-T oligo-attached magnetic beads. Fragmentation was carried out using divalent cations under elevated temperature in NEBNext First Strand Synthesis Reaction Buffer (5X). First strand cDNA was synthesized using random hexamer primer and M-MuLV Reverse Transcriptase (Rnase H-). Second strand cDNA synthesis was subsequently performed using DNA Polymerase I and RNase H. Remaining overhangs were converted into blunt ends via exonuclease/polymerase activities. After adenylation of 3' ends of DNA fragments, NEBNext Adaptor with hairpin loop structure were ligated to prepare for hybridization. In order to select cDNA fragments of preferentially 150~200 bp in length, the library fragments were purified with AMPure XP system (Beckman Coulter, Beverly, USA). Then 3 µl USER Enzyme (NEB, USA) was used with size-selected, adaptor-ligated cDNA at 37°C for 15 min followed by 5 min at 95 °C before PCR. Then PCR was performed with Phusion High-Fidelity DNA polymerase, Universal PCR primers and Index (X) Primer. At last, PCR products were purified (AMPure XP system) and library quality was assessed on the Agilent Bioanalyzer 2100 system.
(3) Clustering and sequencing: The clustering of the index-coded samples was performed on a cBot Cluster Generation System using TruSeq PE Cluster Kit v3-cBot-HS (Illumia). After cluster generation, the library preparations were sequenced on an Illumina Hiseq platform and 125 bp/150 bp paired-end reads were generated.
(4) Quality control: Raw data (raw reads) of fastq format were firstly processed through in-house perl scripts. In this step, clean data (clean reads) were obtained by removing reads containing adapter, reads containing ploy-N and low-quality reads from raw data. At the same time, Q20, Q30 and GC content the clean data were calculated. All the downstream analyses were based on the clean data with high quality.
(5) Reads mapping to the reference genome: Reference genome and gene model annotation files were downloaded from genome website directly. Index of the reference genome was built using STAR and paired-end clean reads were aligned to the reference genome using STAR (v2.5.1b). STAR used the method of Maximal Mappable Prefix (MMP) which can generate a precise mapping result for junction reads.
(6) Quantification of gene expression level: HTSeq v0.6.0 was used to count the reads numbers mapped to each gene. And then FPKM (expected number of Fragments Per Kilobase of transcript sequence per Millions base pairs sequenced) of each gene was calculated based on the length of the gene and reads count mapped to this gene.

2.Detection of IDH mutation

Mutational hotspots of IDH1/IDH2 were evaluated by direct sequencing. Tissues from representative tumor area (the proportion of tumor cells > 20%) were scrapped off from dewaxed sections and treated with PCR reaction solution A 10μl (reaction mixture containing 1μl of cell lysate, 0.3mM of each dNTP, 2.5mM MgCl2, 0.3μM of each primer and 0.2U of KAPA HiFi HotStart DNA Polymerase (Kapa Biosystems Inc., Wilmington, USA)), Shrimp Alkaline Phosphatase (SAP) enzyme (NEB, Ipswich, MA, USA) 2μl and BigDye (BigDye™ Terminator v3.1 Cycle Sequencing Kit, Thermo Fisher Scientific, Waltham, MA, USA) 1μl for centrifugation at 2000 rpm for 10 sec. The crude cell lysate was centrifuged and supernatant was used for subsequent PCR analysis. The forward primer primers (IDH1-F:5’-CGGTCTTCAGAGAAGCCATT-3’,IDH1-R:5’-CACATTATTGCCAACATGAC-3’,IDH2-F:5’-AGCCCATCATCTGCAAAAAC-3’,IDH2-R:5’-CTAGGCGAGGAGCTCCAGT-3’) were used to amplify the region of mutational hotspots of IDH1/IDH2. ①PCR was performed was initiated at 95°C for 5 min, followed by 40 cycles of 95°C for 20 sec, 57°C for 30 sec and 72°C for 1min, and a final extension of 72°C for 5 min and 10°C for 10 min. ②5μl PCR products were then mixed with 2μl SAP enzyme and reacted at 37°C for 40min and then at 80° C for 15min. ③Then 18μl PCR reaction solution C（CWBIO, Beijing, Chima）, 1μl products from ② step, and 1μl BigDye were mixed and reacted at 96°C for 1 min, followed by 30 cycles of 96°C for 10 sec, 50° C for 5 sec and 60° C for 2 min, and a final extension of 25°C for 1 min and 10°C for 10 min. Then 50μl natrium asceticism-ethanol mixture (3M NaAc: ethanol=1:15) were added and the mixture was centrifuged for 30min (12000 rpm, 4°C), with the supernatant being discarded. Then 70μl 75% ethanol were added and the mixture was centrifugated for 15min (12000 rpm, 4°C), with the supernatant being discarded. After complete volatilization of the ethanol at room temperature, 12μl Hi-Di™ Formamide (Thermo Fisher Scientific, Waltham, MA, USA) were added into the precipitate to dissolve the DNA. The dissolved products were sequenced on Applied Biosystems™ 3500DxGenetic Analyzer (Thermo Fisher Scientific, Waltham, MA, USA), and analyzed by Chromas software (Technelysium, South Brisbane, Australia). The sequencing results were compared with wild-type sequences of IDH1/IDH2 for analysis.

3.Detection of chromosome 1p/19q status by Fluorescence in Situ Hybridization (FISH)

Chromosome 1p/19q status was examined by fluorescence in situ hybridization. 4μm thick FFPE sections were baked at 65°C for 2-3h and deparaffinized in xylene for 10 minutes for 2 times. The sections were hydrated by 100％ethanol for 2 min, 85％ethanol for 2 min and 70％ethanol for 2 min orderly, and then immerged in deionized water for 3 min. The sections were processed with citrate repair solutionin (pH6.0) for 4 min in high pressure condition, and then rinsed in 2×SSC solution for 5 min for 2 times. The sections were immerged in protease K fluid（200μg/ml）and incubated for 2 min at 37°C, and then rinsed in 2×SSC solution for 5 min for 2 times. 10μl probes (GP Medical Technologies, Beijing, China) mixture was added to thehybridization zone of the section, and the denaturation and hybridization process was carried out on the ThermoBrite®hybridization instrument (Leica Biosystems, Nussloch, Germany), with denaturation temperature at 83°C for 5 min and hybridization temperature at 42°C for 16h. Sections were immerged in 0.4×SSC plus 0.3% NP-40 cleaning solution (65±1°C) and vibrated for 3 sec. Sections were then retrieved 2 min later and put into 0.1% NP-40 plus 2×SSC cleaning gain/chromosome 10 loss status was examined by fluorescence in situ hybridization. 4μm thick FFPE sections were baked at 65°C for 2-3h and deparaffinized in xylene for 10 minutes for 2 times. The sections were hydrated by 100％ethanol for 2 min, 85％ethanol for 2 min and 70％ethanol for 2 min orderly, and then immerged in deionized water for 3 min. The sections were processed with citrate repair solutionin (pH6.0) for 4 min in high pressure condition, and then rinsed in 2×SSC solution for 5 min for 2 times. The sections were immerged in protease K fluid（200μg/ml）and incubated for 2 min at 37°C, and then rinsed in 2×SSC solution for 5 min for 2 times. 10μl probes (GP Medical Technologies, Beijing, China) mixture was added to thehybridization zone of the section, and the denaturation and hybridization process was carried out on the ThermoBrite®hybridization instrument (Leica Biosystems, Nussloch, Germany), with denaturation temperature at 83°C for 5 min and hybridization temperature at 42°C for 16h. Sections were immerged in 0.4×SSC plus 0.3% NP-40 cleaning solution (65±1°C) and vibrated for 3 sec. Sections were then retrieved 2 min later and put into 0.1% NP-40 plus 2×SSC cleaning solution at room temperature, vibrated for 3 sec and cleansed for 1 min. Then the sections were immerged in 70% ethanol for 3 min and dried avoiding light at room temperature. 15μl DAPI redyeing agent was added into the hybridization zone of the section, and the section was placed avoiding light for 10 min. At last, the section was placed under the BX51TRF fluorescence microscope (Olympus, Tokyo, Japan) for analysis by expert pathologist (Dr. Wei-wei Wang). Hybridizing signals in at least 100 non-overlapping nuclei were counted.

Chromosome 1p/19q status: The loci interrogated were 1p36.3 (RP11-62M23 labeled red)/1q25.3-q31.1 (RP11-162L13 labeled green) and 19q13.3 (CTD-2571L23 labeled red)/19p12 (RP11-420K14 labeled green). A sample was considered 1p or 19q deleted according to the ratio of number of red signal to green signal. In 1p36 or 19q13, positive loss of heterozygosity (LOH) was determined when the ratio of number of red signal to green signal was less than 0.7.

Table S1. Details of Whole Slide Image Preparations

Hospital	FAHZZU	HPPH
**Stainer**	Tissue-Tek Prisma	Tissue-Tek Prisma
**HE Stain solution**	Baso H-E High Definition Constant Staining Solution	Baso H-E High Definition Constant Staining Solution
**Reagents and Protocols**	Xylene 5min	Xylene 5min
	Xylene 5min	Xylene 5min
	100% Ethanol 2min	Xylene 5min
	100% Ethanol 2min	100% Ethanol 2min
	95% Ethanol 2min	95% Ethanol 2min
	75% Ethanol 2min	75% Ethanol 2min
	Wash Station 2min	Wash Station 2min
	Preincubation solution 40s	Preincubation solution 30s
	Hematoxylin 8~11min (Dynamic adjustment according to the accumulated amount of staining)	Hematoxylin 6min
	Wash Station 3min	Wash Station 2min
	Differentiation 1 2s	Differentiation 1 20s
	Differentiation 2 2s	Differentiation 2 20s
	Wash Station 3min	Wash Station 1min
	Blue promoting 50s	Blue promoting 1min
	Wash Station 3min	Wash Station 1min
	Eosin 1~30s (Dynamic adjustment according to the	Eosin 1min

Hospital	FAHZZU	HPPH
	accumulated amount of staining)	
	Wash Station 1min	Wash Station 10s
	95% Ethanol 1min	100% Ethanol 30s
	100% Ethanol 1min	100% Ethanol 1min
	100% Ethanol 2min	100% Ethanol 1min30s
	Xylene 2min	Xylene 2min
	Xylene 2min	Xylene 2min
	Xylene 2min	Xylene 2min
**Digital Pathology Slide Scanner**	KF-PRO-120-HI	KF-PRO-120-HI
**WSI Scan Parameters**	Scan Type: Routine<br>Sectioning Mode: Automatic<br>Number of Layers Scanned: Single layer<br>Magnification: 20X<br>Field of View Scoring Threshold: 0.17<br>Section Scoring Threshold: 60	Scan Type: Routine<br>Sectioning Mode: Automatic<br>Number of Layers Scanned: Single layer<br>Magnification: 20X<br>Field of View Scoring Threshold: 0.17<br>Section Scoring Threshold: 60

Table S2. Details of CellProfiler modules

**Modules**	**Detailed Meaning**	**Parameters**
UnmixColors	UnmixColors creates separate images per dye stain for histologically stained images.	**Hematoxylin**<br>red absorbance:0.571047<br>green absorbance:0.790295<br>blue absorbance:0.222123<br><br>**Eosin**<br>red absorbance:0.438688<br>green absorbance:0.870694<br>blue absorbance:0.222363
IdentifyPrimaryObjects	IdentifyPrimaryObjects identifies biological objects of interest. It requires grayscale images containing bright objects on a dark background. Incoming images must be 2D (including 2D slices of 3D images);	Typical diameter of objects, in pixel units (Min,Max): 10-30<br>Threshold strategy: Adaptive<br>Threshold method: Otsu<br>Two-class or three-class thresholding: Three classes<br>Assign pixels in the middle intensity class to the foreground or the background: Background<br>Threshold smoothing scale: 2<br>Threshold correction factor: 0.8<br>Lower and upper bounds on threshold: 0.5-1.0<br>Size of adaptive window :50
IdentifySecondaryObject	IdentifySecondaryObjects identifies objects (e.g., cells) using objects identified by another module (e.g., nuclei) as a starting point.	Select the method to identify the secondary objects: Watershed -lmage<br>Threshold strategy: Adaptive<br>Thresholding method: Otsu

		Two-class or three-class thresholding: Three classes<br>ssign pixels in the middle intensity class to the foreground or the background: Foreground<br>Threshold smoothing scale:1.3488<br>Threshold correction factor :0.8<br>Lower and upper bounds on threshold: 0.2-1.0<br>Size of adaptive window: 50
IdentifyTertiaryObjects	IdentifyTertiaryObjects identifies tertiary objects (e.g., cytoplasm) by removing smaller primary objects (e.g., nuclei) from larger secondary objects (e.g., cells), leaving a ring shape.	
MeasureObjectIntensity	MeasureObjectIntensity measures several intensity features for identified objects. Given an image with objects identified (e.g., nuclei or cells), this module extracts intensity features for each object based on one or more corresponding grayscale images. Measurements are recorded for each object.	
MeasureObject IntensityDistribution	MeasureObjectIntensityDistribution measures the spatial distribution of intensities within each object. Given an image with objects identified, this module measures the intensity distribution from each object's center to its boundary within a set of bins, i.e., rings that you specify.	Calculate intensity Zernikes: Magnitudes only<br>Maximum zernike moment:9
MeasureTexture	MeasureTexture measures the degree and nature of textures within images and objects to quantify their roughness and smoothness. This module measures intensity variations in grayscale images. An object or entire image without much texture has a smooth	Enter how many gray levels to measure the texture at:256<br>Texture scale to measure:3

	appearance; an object or image with a lot of texture will appear rough and show a wide variety of pixel intensities.	
MeasureGranularity	MeasureGranularity outputs spectra of size measurements of the textures in the image. Image granularity is a texture measurement that tries to fit a series of structure elements of increasing size into the texture of the image and outputs a spectrum of measures based on how well they fit.	Subsampling factor for granularity measurements :0.75<br>Subsampling factor for background reduction :0.2<br>Radius of structuring element: 30<br>Range of the granular spectrum :16
Measure ObjectNeighbors	MeasureObjectNeighbors calculates how many neighbors each object has and records various properties about the neighbors' relationships, including the percentage of an object's edge pixels that touch a neighbor.	
Measure ObjectSizeShape	MeasureObjectSizeShape measures several area and shape features of identified objects. Given an image with identified objects (e.g., nuclei or cells), this module extracts area and shape features of each one.	
ExportToSpreadsheet	ExportToSpreadsheet exports measurements into one or more files that can be opened in Excel or other spreadsheet programs.	

Table S4. Characteristics of patients in training and internal validation sets

	**Training Set (N=486)**	**Internal Validation Set (N=209)**	**P value**
**Age (Year)**			
Mean (SD)	42.9 (10.3)	43.4 (10.8)	0.748
Median [Min, Max]	43.0 [21.0, 73.0]	43.0 [18.0, 77.0]	
**Gender**			
Female	198 (40.7%)	84 (40.2%)	0.959
Male	288 (59.3%)	125 (59.8%)	
**KPS**			
Mean (SD)	77.5 (12.4)	77.1 (12.9)	0.911
Median [Min, Max]	80.0 [20.0, 100]	80.0 [20.0, 100]	
**Resection**			
No	140 (28.8%)	71 (34.0%)	0.205
Yes	346 (71.2%)	138 (66.0%)	
**Radiation**			

	Training Set (N=486)	Internal Validation Set (N=209)	P value
No	101 (20.8%)	37 (17.7%)	0.407
Yes	385 (79.2%)	172 (82.3%)	
**Chemotherapy**			
No	74 (15.2%)	22 (10.5%)	0.127
Yes	412 (84.8%)	187 (89.5%)	
**Tumor type**			
Astrocytoma	243 (50.0%)	105 (50.2%)	0.999
Oligodendroglioma	243 (50.0%)	104 (49.8%)	
**Status**			
Alive	258 (53.1%)	95 (45.5%)	0.078
Dead	228 (46.9%)	114 (54.5%)	
**OS (Month)**			
Mean (SD)	62.3 (30.1)	58.7 (29.0)	0.078
Median [Min, Max]	57.0 [5.40, 151]	53.1 [6.50, 189]	

Table S5. Results of Shapiro-Wilk test for continuous variables in training and internal validation sets

	Training Set (N = 469)		Internal Validation Set (N = 226)	
**Age**	W = 0.99032	P = 0.002784	W = 0.98908	P = 0.1124
**KPS**	W = 0.83971	P = 0.00000000000000022	W = 0.85474	P = 0.0000000000003448
**OS**	W = 0.97691	P = 0.0000005867	W = 0.93774	P = 0.00000008575

Table S6. List of pathomics features used for modeling

Feature Number	Feature name	Location	Type	Coefficient
PF_1	K0 Median Cytoplasm Intensity MADIntensity Eosin	Cytoplasm	Intensity	0.101761992
PF_2	K0 Median Cytoplasm RadialDistribution MeanFrac Eosin 1of4	Cytoplasm	RadialDistribution	0.105480384
PF_3	K0 Median Cytoplasm Texture Correlation Hematoxylin 3 00 256	Cytoplasm	Texture	-0.01209217
PF_4	K0 Median Nuclei AreaShape Zernike 4 2	Nuclei	AreaShape	0.204556729
PF_5	K0 Median Nuclei AreaShape Zernike 9 9	Nuclei	AreaShape	-0.048914718
PF_6	K0 StDev Cytoplasm Granularity 12 Eosin	Cytoplasm	Granularity	0.016637233
PF_7	K0 StDev Cytoplasm Intensity MedianIntensity Eosin	Cytoplasm	Intensity	-0.139026001
PF_8	K0 StDev Nuclei Granularity 10 Hematoxylin	Nuclei	Granularity	0.023540609
PF_9	K0 StDev Nuclei Granularity 12 Eosin	Nuclei	Granularity	0.052225278
PF_10	K0 StDev Nuclei Intensity StdIntensity Hematoxylin	Nuclei	Intensity	0.074940661
PF_11	K0 StDev Nuclei RadialDistribution MeanFrac Eosin 1of4	Nuclei	RadialDistribution	-9.3543E-05
PF_12	K0 StDev Nuclei RadialDistribution MeanFrac Eosin 2of4	Nuclei	RadialDistribution	-0.146621199
PF_13	K0 StDev Nuclei RadialDistribution ZernikeMagnitude Hematoxylin 0 0	Nuclei	RadialDistribution	0.013736387
PF_14	K1 Granularity 13 Eosin	NA	Granularity	0.164508967
PF_15	K1 Granularity 5 Eosin	NA	Granularity	0.029048237
PF_16	K1 Mean Cytoplasm Granularity 13 Eosin	Cytoplasm	Granularity	-0.013236926
PF_17	K1 Mean Nuclei Granularity 9 Eosin	Nuclei	Granularity	0.052331444
PF_18	K1 Median Nuclei AreaShape Zernike 4 4	Nuclei	AreaShape	0.030104684

PF_19	K1 Median Nuclei AreaShape Zernike 7 1	Nuclei	AreaShape	-0.146503525
PF_20	K1 Median Nuclei Granularity 9 Hematoxylin	Nuclei	Granularity	-0.086408691
PF_21	K1 StDev Cytoplasm RadialDistribution MeanFrac Hematoxylin 3of4	Cytoplasm	RadialDistribution	0.130796975
PF_22	K1 StDev Nuclei Texture DifferenceEntropy Eosin 3 00 256	Nuclei	Texture	0.073448362
PF_23	K1 StDev Nuclei Texture DifferenceEntropy Eosin 3 01 256	Nuclei	Texture	0.028264273
PF_24	K2 Mean Nuclei AreaShape Zernike 5 5	Nuclei	AreaShape	-0.065028652
PF_25	K2 Median Cytoplasm Granularity 8 Eosin	Cytoplasm	Granularity	-0.135121512
PF_26	K2 Median Nuclei AreaShape Zernike 4 4	Nuclei	AreaShape	0.144697121
PF_27	K2 Median Nuclei AreaShape Zernike 7 7	Nuclei	AreaShape	-0.038212475
PF_28	K2 StDev Cytoplasm Granularity 11 Hematoxylin	Cytoplasm	Granularity	0.146463487
PF_29	K2 StDev Cytoplasm Granularity 12 Eosin	Cytoplasm	Granularity	0.127746815
PF_30	K2 StDev Cytoplasm Granularity 6 Eosin	Cytoplasm	Granularity	0.049188467
PF_31	K2 StDev Cytoplasm Granularity 8 Hematoxylin	Cytoplasm	Granularity	0.13198625
PF_32	K2 StDev Cytoplasm Intensity MADIntensity Hematoxylin	Cytoplasm	Intensity	0.088116248
PF_33	K2 StDev Cytoplasm Intensity MeanIntensity Eosin	Cytoplasm	Intensity	-8.3769E-05
PF_34	K2 StDev Cytoplasm RadialDistribution ZernikeMagnitude Eosin 0 0	Cytoplasm	RadialDistribution	-0.025123629
PF_35	K2 StDev Cytoplasm RadialDistribution ZernikeMagnitude Eosin 9 3	Cytoplasm	RadialDistribution	-0.175515474

Table S7. Cox regression analysis within the training, internal validation and external validation sets

Variables	Univariate analysis		Multivariate analysis	
	HR (95% CI)	P-value	HR (95% CI)	P-value
		Training Set		
Age*	1.022 (1.008~1.035)	0.001	1.020 (1.006~1.034)	0.004
Gender	0.951 (0.731~1.238)	0.711	——	——
KPS	1.009 (0.998~1.020)	0.118	——	——
Resection*	0.644 (0.487~0.852)	0.002	0.680 (0.513~0.904)	0.008
Radiation*	0.604 (0.449~0.813)	0.000	0.766 (0.562~1.043)	0.090
Chemotherapy	0.840 (0.597~1.182)	0.317	——	——
Tumor type*	0.463 (0.354~0.605)	0.000	0.480 (0.363~0.635)	0.000
Pathscore*	3.707 (2.963 ~ 4.638)	0.000	3.075 (2.437~3.880)	0.000
		Internal Validation Set		
Age	1.009 (0.990~1.028)	0.346	——	——
Gender	0.879 (0.605~1.277)	0.498	——	——
KPS	0.996 (0.982~1.010)	0.595	——	——

Variables	Univariate analysis		Multivariate analysis	
	HR (95% CI)	P-value	HR (95% CI)	P-value
Resection	0.910 (0.607~1.366)	0.649	——	——
Radiation	0.899 (0.572~1.413)	0.645	——	——
Chemotherapy	0.655 (0.386~1.113)	0.118	——	——
Tumor type*	0.620 (0.427~0.902)	0.012	0.616 (0.424~0.894)	0.011
Pathscore*	1.415 (1.009~1.983)	0.044	1.416 (1.019~1.967)	0.039
HPPH Validation Set				
Age	1.008 (0.977~1.039)	0.615	——	——
Gender	0.780 (0.381~1.600)	0.498	——	——
KPS	0.995 (0.959~1.032)	0.795	——	——
Resection	0.570 (0.244~1.333)	0.194	——	——
Radiation	1.213 (0.554~2.654)	0.629	——	——
Chemotherapy	1.500 (0.521~4.317)	0.453	——	——
Tumor type*	0.455 (0.220~0.940)	0.033	0.398 (0.190~0.835)	0.015
Pathscore*	2.351 (1.129~4.898)	0.022	2.540 (1.246~5.175)	0.010
TCGA Validation Set				
Age*	1.036 (1.013~1.059)	0.002	1.035 (1.013~1.058)	0.002

Variables	Univariate analysis		Multivariate analysis	
	HR (95% CI)	P-value	HR (95% CI)	P-value
Gender	0.977 (0.574~1.663)	0.932	——	——
Race black or African American	0.340 (0.030~3.811)	0.381	——	——
Race not reported	0.514 (0.032~8.273)	0.639	——	——
Race white	0.300 (0.040~2.224)	0.239	——	——
Histology Mixed glioma	0.678 (0.335~1.373)	0.280	——	——
Histology Oligodendroglioma	0.654 (0.355~1.208)	0.175	——	——
Pathscore*	1.393 (1.064~1.823)	0.016	1.417 (1.070~1.877)	0.015

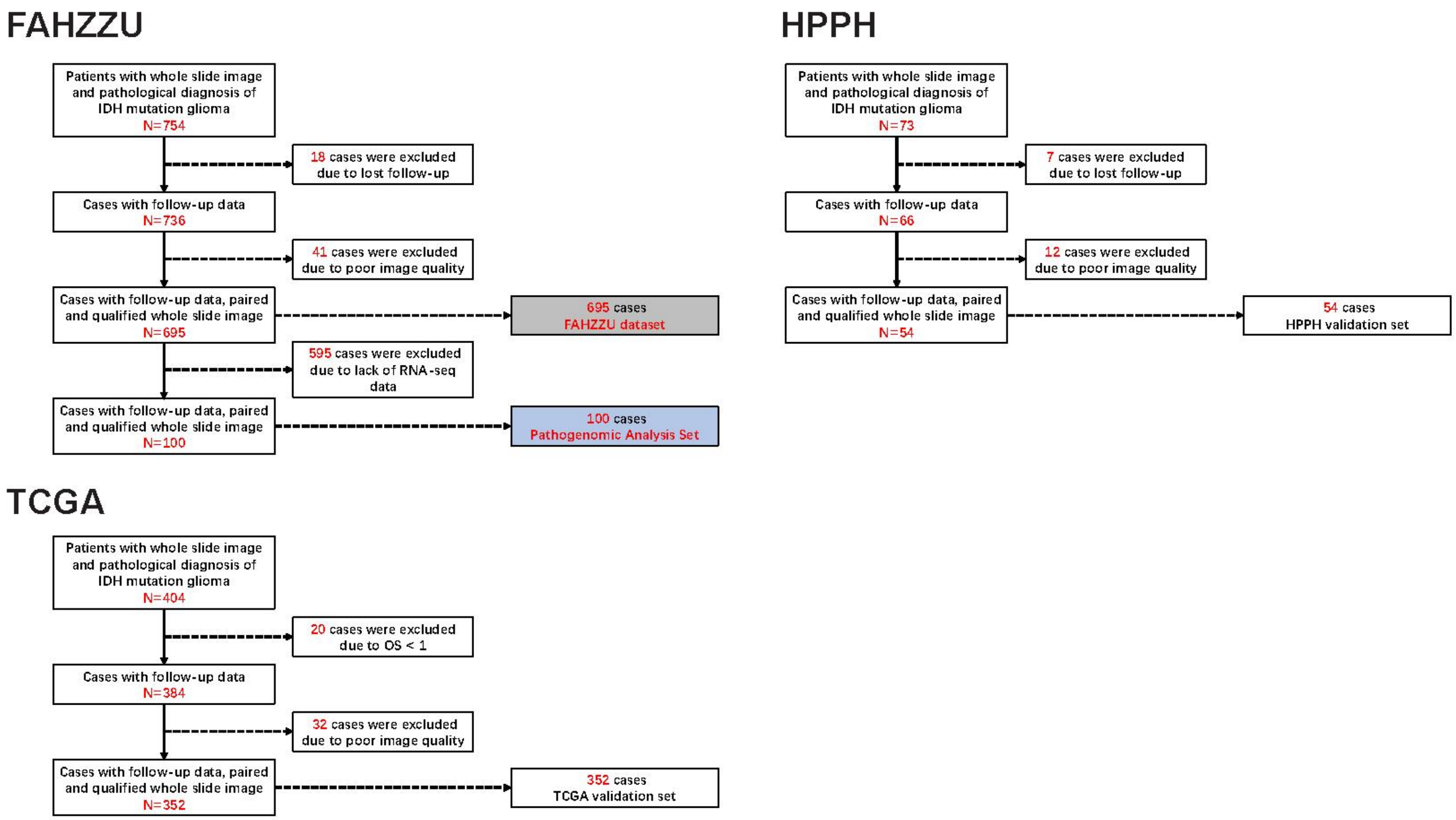

Figure S1. The criteria for patients' inclusion and exclusion

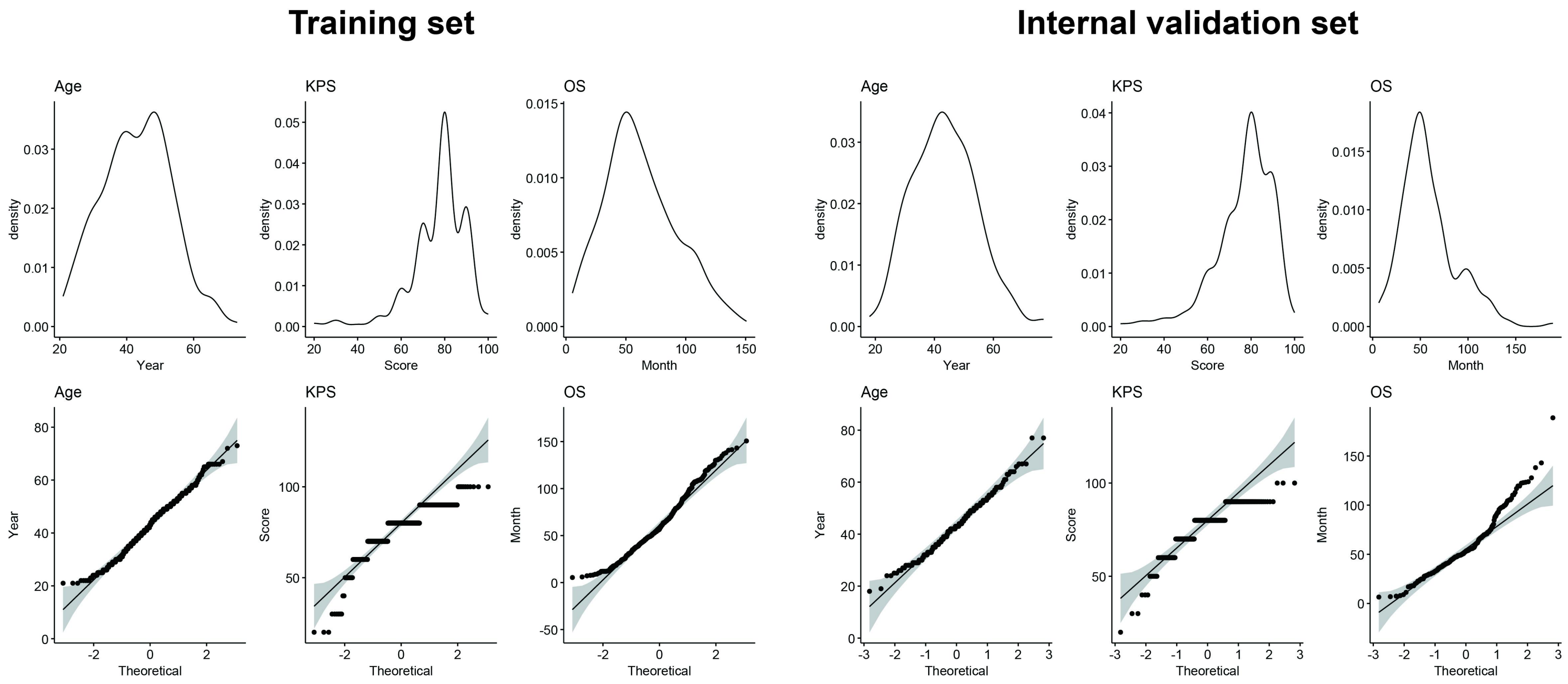

Figure S2. Continuous variable normal test visual results

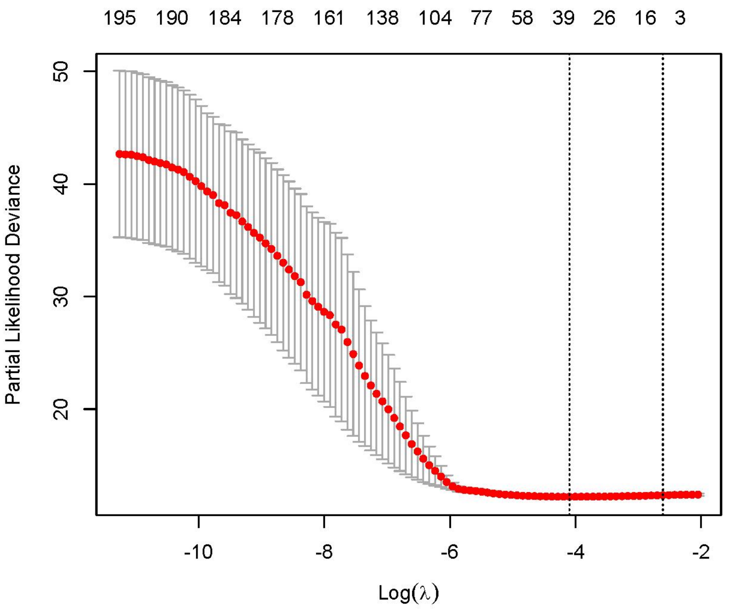

Figure S3. Pathomics feature selection 1

Pathomics feature selection by using the LASSO Cox model. Ten-fold cross validation is used to determine the optimal LASSO parameter λ. According to parameter λ, the coefficients of the most irrelevant features were shrunk to zero.

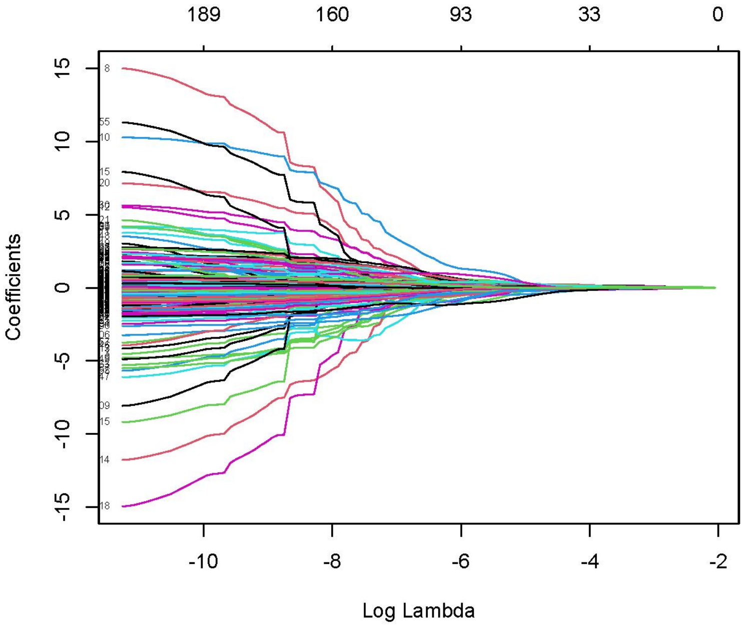

Figure S4. Pathomics feature selection 2

Pathomics feature selection by using the LASSO Cox model. The partial likelihood deviance was plotted versus log(λ).

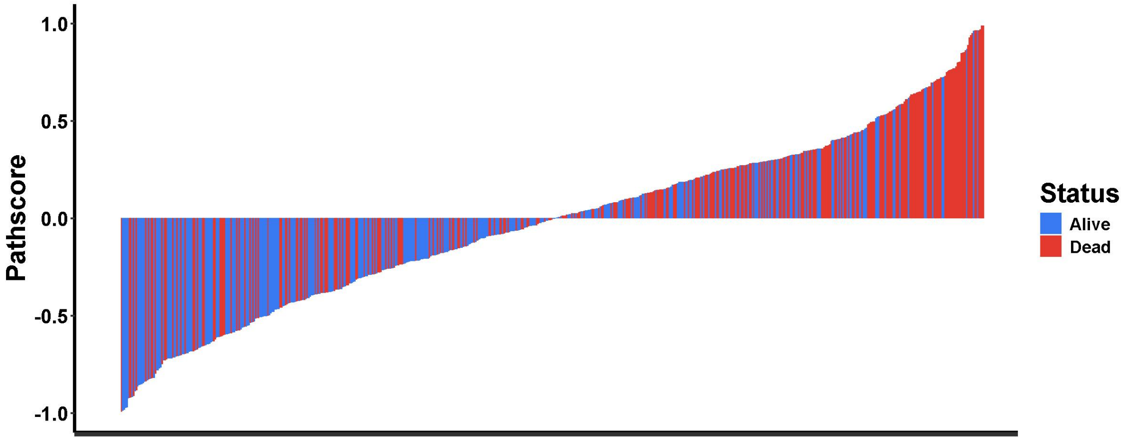

Figure S5. Pathscore for each patient

Based on the cutoff value of 0.09472189 generated by R package survminer, patients were divided into a high-risk group (Pathscore ≥ 0.1289816) and a low-risk group (Pathscore ≤ 0.1289816). The status of dead or censorship was marked with different colors.
